# Voice identity invariance by anterior temporal lobe neurons

**DOI:** 10.1126/sciadv.adv7033

**Published:** 2025-08-29

**Authors:** Margherita Giamundo, Régis Trapeau, Etienne Thoret, Luc Renaud, Thomas Brochier, Pascal Belin

**Affiliations:** ^1^ Institut de Neurosciences de la Timone, UMR 7289, CNRS, Aix-Marseille Université, Marseille 13005, France.; ^2^ Institute of Language Communication and the Brain, ILCB, Aix-en-Provence 13100, France.

## Abstract

The ability to recognize speakers by their voice despite acoustical variation plays a substantial role in primate social interactions. Although neurons in the macaque anterior temporal lobe (ATL) show invariance to face viewpoint, whether they also encode abstract representations of caller identity is not known. Here, we demonstrate that neurons in the voice-selective ATL of two macaques support invariant voice identity representations through dynamic population-level coding. These representations minimize neural distances across different vocalizations from the same individual while preserving distinct trajectories for different callers. This structure emerged from the coordinated activity of the broader neuronal ensemble, although a small subset of highly identity-selective neurons carried high identity information. Our findings provide a neural basis for voice identity recognition in primates and highlight the ATL as a key hub for integrating perceptual voice features into higher-level identity representations.

## Teaser

Neurons in the macaque temporal lobe recognize callers despite acoustical variation.

## INTRODUCTION

Human listeners readily extract speaker identity information from even brief voice samples—an ability at the core of our social interactions. Nonhuman primates share a similar ability as shown by both field and laboratory studies (*1*–*4*). Although neuroimaging studies start shedding light on the neural circuits involved in voice processing by humans, an understanding of how identity information is encoded at the level of individual neurons is still lacking, in contrast to face identity processing (*5*–*8*).

Numerous studies in humans and nonhuman primates suggest that a key biological substrate for identity processing is the anterior temporal lobe (ATL) as a convergence site for different sensory streams [for reviews, see (*9*–*12*)]. In the auditory modality, the anterior temporal voice area (aTVA) is an ATL region located downstream from primary auditory cortex (A1) along the auditory object recognition (“what”) ventral stream and specialized in the processing of conspecific vocalizations in humans and macaques (*13*–*17*).

Functional magnetic resonance imaging (fMRI) studies found that the aTVA, but not A1, shows adaptation to speaker identity in both humans (*18*) and macaques (*13*), i.e., decreases its neural activity when different consecutive stimuli are from a same speaker, suggesting abstract representation of speaker identity (*19*–*21*). How such an abstract, variability-robust representation of speakers emerges at the level of individual neurons is still largely unknown. A single study, thus far, has investigated the processing of caller identity in macaque aTVA neurons, showing that a small subset of these neurons differentiates between individuals more than between call types (*22*), but whether and how they are involved in caller recognition is unclear.

Here, we investigate to what extent neural population dynamics in the macaque aTVA process voice identity information. As suggested by electrophysiological studies of face identity processing (*6*, *7*), we hypothesize that voice identities are represented in an abstract, distributed manner across the broader neuronal population, enabling robust recognition of individuals despite natural acoustic variability. An alternative, nonmutually exclusive hypothesis is that a small subset of highly selective, sparsely active neurons—similar to the “grandmother neurons” observed in the medial temporal lobe (*23*)—may carry disproportionately strong identity information by responding selectively to individual callers.

To test these hypotheses, we used fMRI-guided electrophysiological recordings in two macaques to examine neural activity in the aTVA during exposure to vocalizations from five distinct conspecific callers. Our findings reveal that (i) aTVA neuronal populations encode voice identity with notable accuracy; (ii) this identity representation emerges from a distributed population code, rather than relying solely on a small subset of highly selective neurons; and (iii) aTVA neurons support caller recognition by minimizing neural distances between different vocalizations produced by the same individual, thus enabling abstraction across acoustic variability.

## RESULTS

**Fig. 1.**
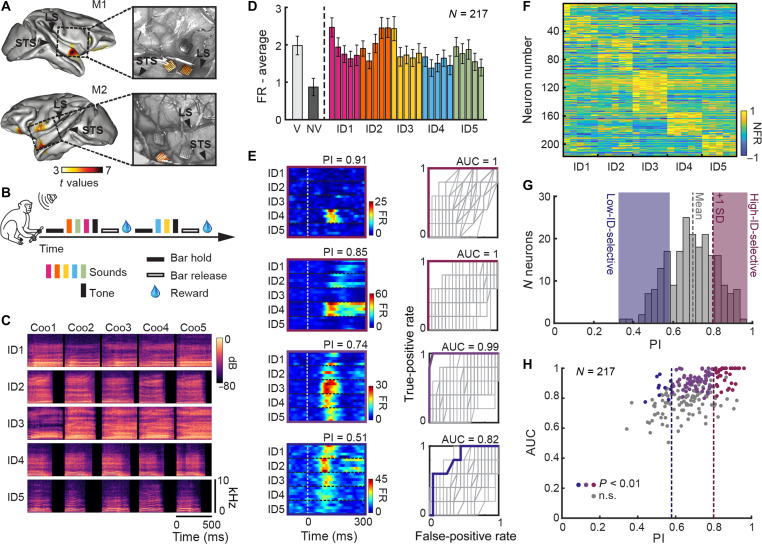
aTVA neuronal activity is modulated by voice identity. (**A**) Implantation sites of Utah Arrays in monkeys M1 and M2. Color scale indicates *t* values of fMRI contrast between conspecific macaque vocalizations versus nonvocal sounds. Arrays analyzed here (highlighted in pictures of cortical surface during surgery, right insets) were placed in cortical areas close to the fMRI-identified aTVA peaks. STS, superior temporal sulcus; LS, lateral sulcus. (**B**) Monkeys were trained to release a bar when a pure tone was randomly presented amongst other stimuli to obtain the reward. (**C**) Spectrograms of the 25 “coo” calls produced by five individual macaques (IDs). (**D**) Average population responses (means ± SE) to macaque vocalizations (V) and nonvocal sounds (NV) of the localizer and to the 25 voice identity (ID) stimuli, with the color corresponding to each identity. (**E**) Response time courses to the 25 stimuli (left) and corresponding ROC curves (right; color traces) of four representative neurons. Gray curves show 99 surrogate ROC curves computed from randomly selected voice sets. (**F**) Neurons responsiveness to the 25 stimuli. Each row represents the average response of a single neuron in the 200-ms window following stimulus onset. Neurons are sorted on the basis of the identity eliciting their strongest response. NFR, normalized firing rate. (**G**) Histogram of PI values, reflecting each neuron’s selectivity for a single identity. Shaded areas highlight two subgroups: High-ID-selective neurons (magenta), showing strong preference for one identity, and Low-ID-selective neurons (blue), showing weaker selectivity. (**H**) Distribution of PI and ROC AUC values for all neurons. Colored dots indicate neurons with significant AUC values (*P* < 0.01) for the High-ID-selective group (34 of 44 neurons, 77%; magenta), the Low-ID-selective group (11 of 44, 25%; blue), and the intermediate group—i.e., neurons not in the extreme PI categories (69 of 129, 53%; violet). n.s., not significant.

To investigate the neural basis of voice identity processing, we recorded spiking activity from single neurons of two female rhesus monkeys (M1 and M2) implanted with chronic multielectrode Utah arrays in the fMRI-localized aTVA (Fig. 1A) (*16*, *17*). The aTVA was functionally localized in each animal using a previously established fMRI voice localizer, which identified regions that responded more strongly to conspecific vocalizations than to nonvocal sounds. M1 was implanted with two 32-channel Utah arrays targeting the aTVA in the right rostral superior temporal gyrus (rSTG), whereas M2 was implanted with one 32-channel array targeting the left rSTG. These recording sites were located in hierarchically high-level auditory cortex near the temporal pole and lacked clear tonotopic organization (*17*).

To ensure sustained auditory attention, the electrophysiological recordings took place while the monkeys performed active detection of a pure tone interspersed amongst a set of complex vocal stimuli (Fig. 1B). The set of stimuli consisted of 25 natural vocalizations: five distinct “coo calls” from each of the five unfamiliar macaque individuals (Fig. 1C). Coo calls, also termed “contact calls,” are harmonically rich affiliative vocalizations commonly produced by macaque species in social contexts [see (*4*) for a review]. We chose to concentrate on this class of vocalizations due to macaques’ demonstrated ability to recognize individual conspecifics by their coo calls (*24*).

We focused our analyses on 217 well-isolated auditory-responsive neurons (173 of 358 from M1 and 44 of 117 from M2), i.e., neurons responding to at least 1 of the 25 stimuli (see Materials and Methods). Below, we combine the data from the two monkeys because we did not find any marked differences between individuals, but see figs. S1 to S4 for the main results separately for each animal.

### aTVA neurons exhibit strong identity selectivity

We initially assessed the selectivity of neurons to voices by comparing their spiking activity in response to the 25 voice identity stimuli with their responses to a localizer consisting of 12 macaque vocalizations and 12 nonvocal sounds presented at the beginning of each recording session (see Materials and Methods). The aTVA neuronal population exhibited a stronger response to macaque vocalizations compared to nonvocal sounds (Fig. 1D and fig. S1, A and B), confirming the voice-sensitive nature of this region.

To quantify this selectivity, we computed a voice selectivity index (VSI) for each neuron, by contrasting firing rates (FRs) in response to the 12 macaque vocalizations and the 12 nonvocal sounds of the localizer (see Materials and Methods). Using a criterion of VSI ≥ 0.33 (indicating at least twice the response to vocalizations compared to nonvocal sounds), 42% of neurons (92 of 217) were classified as voice-selective (fig. S1D). When also including neurons that were selectively inhibited by vocalizations (VSI ≤ −0.33), as commonly done in the face-selectivity literature [e.g., (*25*)], the proportion of voice-selective neurons increased to 70% (152 of 217).

Although the average spiking activity of the aTVA neuronal population did not show substantial differences across the five macaque identities (Fig. 1D and fig. S1B), individual neurons exhibited diverse response patterns, ranging from broad responses to multiple identities to highly specific responses for a single identity (Fig. 1, E and F, and figs. S1C and S2A). We quantified the selectivity of neurons to one identity using a preference index (PI; Fig. 1G and fig. S1, E and F). The PI measures a neuron’s relative response to its preferred identity versus others, with values near 1 indicating strong selectivity for a specific identity, and values near 0 indicating similar responses to all five identities. Overall, aTVA neurons exhibited strong identity selectivity with a mean PI of 0.7 (± 0.1 SD), supporting the role of this region in encoding voice identity. Notably, identity selectivity (PI) was not significantly correlated with voice selectivity (VSI; Pearson correlation, rho = 0.09, *P* = 0.18).

To assess invariance in responses across vocalizations of the same individual, we implemented a receiver operating characteristic (ROC) analysis (*23*, *26*). Neurons that responded similarly to all vocalizations from one identity but not to others exhibited convex ROC curves with area under the curve (AUC) values approaching 1. In contrast, neurons with inconsistent responses exhibited AUCs near 0.5, reflecting chance-level discrimination (Fig. 1E and fig. S2B). A neuron was considered to have an invariant representation if its AUC exceeded the values of 99 surrogate curves (*P* < 0.01) generated by testing responses to randomly selected sets of voices (Fig. 1E and fig. S2B, gray curves). On the basis of this criterion, 53% of neurons (114 of 217) exhibited invariant coding for the preferred identity, including four neurons that were also selective for an additional identity (fig. S2C). Moreover, the AUC-based invariance measure was significantly correlated with identity selectivity (PI) (Pearson’s rho = 0.52, *P* < 0.001; Fig. 1H), indicating that neurons with stronger identity preference also generalized more robustly across different vocalizations of the same individual.

### Voice identity is encoded at the population level

**Fig. 2.**
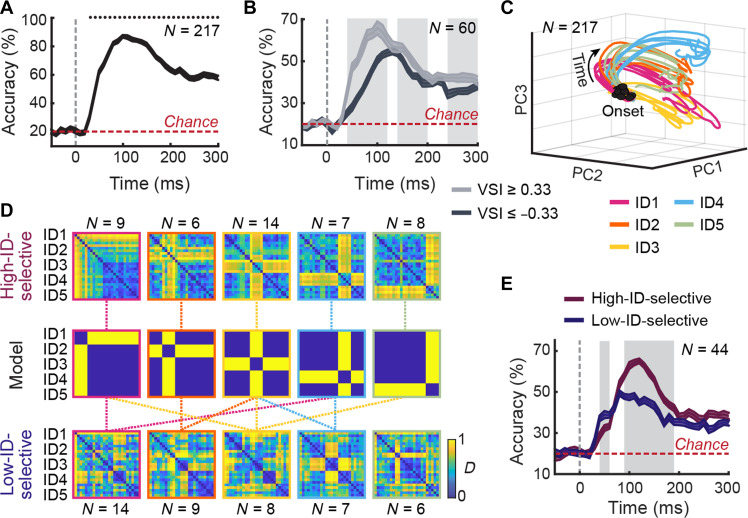
Voice identity is encoded through distributed and time-resolved population dynamics. (**A**) Time-resolved classification accuracy (means ± SE) of a linear classifier trained to discriminate between the five voice identities (chance level = 20%) based on population spiking activity. Black dots indicate time bins with significantly above-chance classification accuracy (permutation tests, *P* < 0.0004). (**B**) Identity classification accuracy for neurons selectively activated (VSI ≥ 0.33) or selectively inhibited (VSI ≤ −0.33) by voices. Gray shaded areas indicate time bins with a significant difference between the two subpopulations (η < 0.05). (**C**) Neural response trajectories for the 25 stimuli projected into a low-dimensional space defined by the first three PCs, from −100 to +300 ms relative to stimulus onset. (**D**) Neuronal RDMs computed from high-identity-selective (top) and low-identity-selective (bottom) neurons, grouped by preferred identity. Color scales indicate normalized pairwise distance rankings. Middle: Corresponding theoretical Model RDMs, each representing ideal distinction of a single identity from all others. Dashed lines indicate significant comparisons between Neuronal and Model RDMs (bootstrapped two-sample *t* tests, Bonferroni-corrected *P* < 0.001). (**E**) Identity classification accuracy for high-identity-selective and low-identity-selective neurons. Gray shaded areas indicate time bins with a significant difference between the two subpopulations (η < 0.05).

We investigated the role of the aTVA neuronal population in encoding voice identity by training a machine learning classifier [maximum correlation coefficient (MCC)] to distinguish between identities based on spiking activity (20-ms time windows every 10-ms steps; Fig. 2A and fig. S3A). Decoding accuracy was significantly above chance (chance = 20%) from 30 ms after stimulus onset and persisted throughout the entire duration of the sound (all *P* values < 0.0004, permutation tests), reaching 88% of accuracy at 100 ms after onset (Fig. 2A). The classifier performed consistently across all identities, with no substantial differences in misclassification rates (fig. S3B).

To examine whether voice selectivity influences identity encoding, we conducted the same decoding analysis specifically on voice-selective neurons (Fig. 2B). We compared the classification performance of neurons selectively activated by voices (i.e., neurons with VSI ≥ 0.33) to that of neurons selectively inhibited (i.e., VSI ≤ −0.33). For reference, decoding results based on a broader classification of neurons grouped by different ranges of VSI values are shown in fig. S3C, providing a more fine-grained view of how varying degrees of voice selectivity impact identity classification. Although identity classification accuracy was higher for neurons selectively activated by voices (40 to 120, 140 to 200, and 240 to 300 ms; all η values < 0.05), both subpopulations achieved significantly above-chance classification performance starting at 40-ms poststimulus onset (all *P* values < 0.0004, permutation tests).

To visualize the dynamics of the neuronal population’s responses, we applied a dimensionality reduction approach by principal components analysis (PCA). This allowed us to represent the responses to the 25 vocalizations as neural trajectories evolving in a low-dimensional space. Figure 2C shows the neural trajectories across the stimulus presentation time, plotted in the subspace defined by the first three principal components (explained variance: PC1 = 15%, PC2 = 13%, and PC3 = 8%). Following stimulus onset, trajectories rapidly converged for vocalizations from the same identity and diverged for different identities in the low-dimensional space. To quantify this pattern, we computed the Euclidean distances between neural trajectories, revealing a significant increase in the distance of trajectories between identities compared to the distance of trajectories within each identity from stimulus onset (from −4 ms throughout the stimulus presentation time, all *t*(298) > 3.7905 and all *P* values below Bonferroni-corrected *P* value = 0.0001). These findings show that the aTVA population robustly encodes voice identity through dynamic and distinct neural patterns.

### Voice identity emerges from distributed neural coding rather than from a few highly selective neurons

We observed that many neurons exhibited strong selectivity for specific individuals (Fig. 1E and fig. S2A), raising the question of whether voice identity information is distributed across the entire neuronal population or whether a smaller subset of highly identity-selective neurons carries sufficient information to support individual recognition. To address this, we compared the contributions of high-identity-selective neurons to those of more broadly tuned neurons. Neurons with a PI greater than 0.8 (i.e., 1 SD above the mean) were classified as high-identity-selective (*N* = 44; Fig. 1G). These neurons varied in their preferred identity, determined by their tuning profiles, and were differently distributed across the recording arrays (fig. S4A). For comparison, we selected a matched group of low-identity-selective neurons (*N* = 44) with the lowest PI values (mean PI ± SD = 0.52 ± 0.05; Fig. 1G).

To examine how these subpopulations represent voice identity, we applied representational similarity analysis (RSA) (*27*), which quantifies the dissimilarity in population spiking activity—measured as Euclidean distance in multineuron space—between each pair of stimuli. This allowed us to test whether selected neurons encoded a specific identity consistently across different vocalizations. For this analysis, neurons in each subpopulation were grouped by their preferred identity, and for each group, we computed a 25 × 25 neuronal representational dissimilarity matrix (RDM) using spike data from the first 200 ms after stimulus onset. This resulted in five RDMs for high-identity-selective neurons (Fig. 2D, top) and five RDMs for low-identity-selective neurons (Fig. 2D, bottom). These were compared to five theoretical Model RDMs, each reflecting an idealized identity representation: minimal dissimilarity between calls from the same individual and maximal dissimilarity from all others (Fig. 2D, middle). RDMs from high-identity-selective neurons closely matched their corresponding Model RDMs, whereas RDMs from low-identity-selective neurons showed weaker and more inconsistent alignment (bootstrapped two-sample *t* tests, Bonferroni-corrected *P* < 0.001; Fig. 2D).

We next used the same decoding approach applied to the full population (Fig. 2A) to test identity classification in each subpopulation. Both high- and low-identity-selective subpopulations performed significantly above chance (from the 30-ms poststimulus onset for low-identity-selective neurons and 40 ms for high-identity-selective neurons, all *P* values < 0.0004, permutation tests; Fig. 2E), although they differed in temporal dynamics and accuracy (η values < 0.05). High-identity-selective neurons achieved higher classification accuracy, peaking at the 120-ms poststimulus onset, albeit with a slightly delayed onset. Conversely, low-identity-selective neurons demonstrated faster but less accurate classification, peaking at 80 ms (Fig. 2E). Despite these differences, both subpopulations demonstrated similar temporal coding stability, with decoding accuracy highest when training and testing occurred at the same time point, suggesting that voice identity representations evolved dynamically over time (fig. S4, B and C).

Together, these findings suggest that, although highly identity-selective neurons provide more precise information, voice identity recognition is ultimately supported by distributed coding across the broader neuronal population.

### aTVA neural population prioritize voice recognition over discrimination

Voice identity processing in humans has been proposed to consist of two main abilities (*28*–*30*): (i) Discrimination: perceiving that two voice samples are from separate individuals (“telling voices apart”), primarily based on low-level acoustical differences; and (ii) Recognition: perceiving that two voice samples have been produced by a same individual (“telling voices together”), a higher-level ability requiring abstraction from within-speaker acoustical variability. Unraveling how these dual processes occur in populations of individual neurons is crucial for understanding the neural mechanisms underlying voice identity processing and more generally, person perception.

**Fig. 3.**
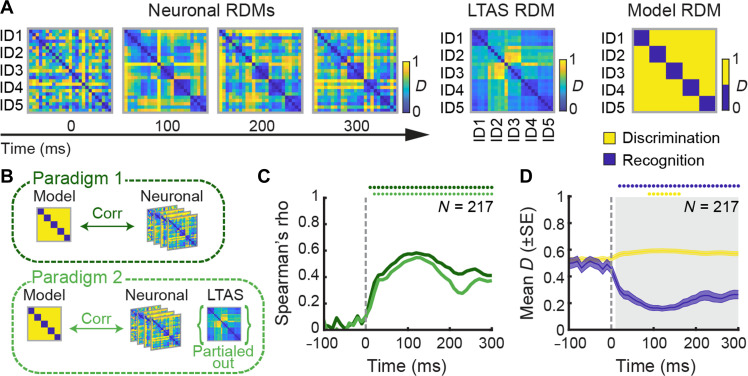
Neural patterns underling Discrimination and Recognition processes. (**A**) The different RDMs compared. Left: Time-resolved Neuronal RDMs capturing spiking differences across all 25 voice identity stimuli. Color scale indicates normalized pairwise distance rankings. Middle: Acoustical RDM based on LTAS. Right: Theoretical Model RDM representing ideal distinction of Discrimination and Recognition patterns. (**B**) Schematic of the two analysis paradigms. (**C**) Time course of correlations (Spearman) derived from Paradigm 1 (dark green curve) and Paradigm 2 (light green curve). Colored dots indicate time bins with significant correlations (Bonferroni-corrected *P* < 0.0012). (**D**) Dissimilarity values (means ± SE) of the portions of Neuronal RDMs corresponding to Discrimination (yellow curve) and Recognition (blue curve) processes, as predicted by the theoretical model. Gray shaded area indicates time bins with significant alignment between Neuronal and Model RDMs (bootstrapped two-sample *t* tests, Bonferroni-corrected *P* = 0.0012). Colored dots indicate time bins with a significant difference from baseline (paired *t* tests, Bonferroni-corrected *P* < 0.0012).

We sought to disentangle the neural patterns underlying these two key components of voice identity processing by applying RSA to time-resolved spiking activity. We computed a 25 × 25 Neuronal RDM every 10 ms during stimulus presentation, using a 50-ms window centered on each time bin (Fig. 3A, left). We then compared these time-resolved Neuronal RDMs to a theoretical Model RDM designed to capture the contribution of both Discrimination and Recognition processes (Fig. 3A, right). The model depicts an ideal pattern of dissimilarities between pairs of stimuli, where voices from the same identity are grouped together, showing no dissimilarity in their neural responses (Recognition, shown in blue), whereas voices from different individuals exhibit maximum dissimilarity (Discrimination, shown in yellow). Because vocalizations from the same individual may share more similar low-level acoustic features than those from different individuals, we also computed an acoustic RDM based on the long-term average spectrum (LTAS) of the vocalizations (Fig. 3A, middle). The LTAS captures differences in spectral shape, encompassing both source-related and filtering characteristics of each sound (*31*).

To assess the correspondence between neural data and these models, we calculated Spearman’s partial rank correlations between the upper triangles (excluding the diagonal) of the Neuronal, Model, and LTAS RDMs at each time bin. We used two analysis paradigms (Fig. 3B): In Paradigm 1, we directly correlated time-resolved Neuronal RDMs with the theoretical Model RDM to evaluate overall correspondence. In Paradigm 2, we repeated the analysis while controlling for the LTAS RDM, allowing us to estimate the variance in neural patterns uniquely attributable to higher-level identity features, by removing the influence of low-level acoustic similarity (*32*).

In Paradigm 1, correlations between Neuronal and Model RDMs were strong, emerging at 10-ms poststimulus onset and persisting throughout the stimulus presentation (Bonferroni-corrected *P* < 0.0012; Fig. 3C). In Paradigm 2, correlations remained significant even after accounting for LTAS, although slightly reduced, starting from 20-ms poststimulus (Bonferroni-corrected *P* < 0.0012). Moreover, direct comparisons revealed that correlations between Neuronal and LTAS RDMs were lower than those between Neuronal and Model RDMs (fig. S5A), indicating that low-level acoustic features only modestly contributed to neural similarity.

To directly assess the contributions of Discrimination and Recognition, we isolated the portions of the Neuronal RDMs corresponding to these processes and compared their dynamics to the Model (Fig. 3D). The model-neural alignment reached significance at 10-ms poststimulus and persisted throughout (bootstrapped two-sample *t* tests, Bonferroni-corrected *P* < 0.0012). Discrimination-related distances—i.e., between voices from different individuals—showed only a modest increase relative to baseline (−100 to 0 ms), becoming significant between 90 and 160 ms (paired *t* tests, Bonferroni-corrected *P* < 0.0012; Fig. 3D, yellow curve). In contrast, Recognition-related distances—i.e., between different vocalizations from the same individual—decreased significantly from 20 ms, with this effect persisting throughout the stimulus duration (paired *t* tests, Bonferroni-corrected *P* < 0.0012; Fig. 3D, blue curve). This pattern was consistent across both high-identity-selective and low-identity-selective neuronal subpopulations, which showed comparable dynamics in representing Recognition and Discrimination (fig. S5B). It is important to note, however, that these two processes are not mutually exclusive: A neuron exhibiting strong Recognition performance also contributed to voices Discrimination (fig. S5, C and D).

Together, these results indicate that the aTVA population primarily supports Recognition by reducing neural distances between different calls from the same individual, enabling abstraction across acoustic variability.

## DISCUSSION

Together, our results show that neurons in the voice-selective ATL encode caller identity via identity-invariant representations, allowing recognition of individuals despite acoustical variations. We recorded the spiking activity of over 200 auditory-responsive neurons in the anterior voice patch of two macaques, individually localized using fMRI. Approximately 70% of these neurons were classified as voice-selective—considerably higher than the 21% initially reported in the seminal “voice cell” study (*14*) and approaching the 97% face-selectivity observed in the anterior face patch (*25*). This discrepancy may reflect improved patch localization or differences in task demands.

Individual neurons showed different response profiles to the five macaque identities presented, from broad response to multiple identities to highly selective response to a single identity. Although the subpopulation of neurons with high-identity-selectivity had more consistent responses to their preferred identity (Figs. 1H and 2D) and yielded the highest identity classification accuracy, a subpopulation of lower-identity-selective neurons also yielded high, if inferior, accuracy (Fig. 2E). This suggests a distributed code involving neurons with multiple patterns of selectivity rather than a sparse code relying on a small number of highly identity-selective “grandmother” cells. Even the neuronal subpopulation with low voice-selectivity yielded far above-chance identity classification accuracy (Fig. 2B), suggesting sound-source recognition mechanisms common to all voice patch neurons and potentially applying to sound sources other than vocalizations.

Collectively, aTVA neurons showed rapid convergence of firing patterns across stimuli from the same caller while preserving distinct trajectories for each identity (Fig. 2C). The representational geometry for the 25 stimuli rapidly converged to a binary Recognition/Discrimination model (max. Spearman rho close to 0.6), even after taking low-level acoustical differences into account (Fig. 3, B and C). Crucially, caller invariance was achieved by a rapid relative decrease in within-identity neuronal distances (Fig. 3D) such that minimal within-identity spiking rate differences were attained around 100 ms after onset. Such caller invariance parallels the viewpoint-invariant responses observed in face-selective neurons. Neurons of the most anterior face patch, the anterior medial (AM; located close to the aTVA within the ATL), fire similarly to images of individual faces despite variations in viewpoints, a phenomenon not observed in more posterior, earlier-stage face patches (*5*). Although it would be tempting to suggest the existence of a similar hierarchy of increasingly abstract voice representations along the macaque temporal lobe (*33*, *34*), the present results are too preliminary: Only five caller identities were used, and a single call type, when natural speaker recognition extends to many more identities, and across call types. Also, no comparison sites at earlier voice processing stages closer to A1 are available, where the face-voice analogy would predict less invariant identity recognition—an important limitation to be addressed by future studies.

A key question for future research is whether aTVA neurons also participate in cross-modal identity representations, similar to the “concept cells” identified in the medial temporal lobe (*35*), integrating sensory information into abstract identity constructs. In this light, it is important to note that audiovisual integration has already been observed by some neurons in the aTVA (*22*) and in the face-processing system (*36*). Future studies should explore whether aTVA neurons also participate in cross-modal integration and whether joint representations of face and voice identity converge at or beyond this level.

Our results align with previous studies in humans and macaques, which consistently underscore the ATL’s critical role in processing high-level cognitive features related to identity (*9*–*12*). The anterior voice patch may represent the final stage in the perceptual analysis of voice features before relaying this information to higher-order associative regions within the temporal lobe and prefrontal cortex. In particular, the middle temporal lobe (*26*) and the temporal pole (*37*) may contribute to the integration of voice features with other identity-related signals, facilitating the formation of new associations, memory-based recognition and the feeling of familiarity [e.g., (*38*, *39*)]. Whereas prefrontal regions such as ventrolateral prefrontal cortex (*40*–*42*) appear to integrate multimodal identity cues with contextual and behavioral information, forming abstract cognitive representations of individuals that guide goal-directed behavior and support social communication.

## MATERIALS AND METHODS

### Experimental design

#### Subjects

Data were recorded from two female rhesus monkeys (*Macaca mulatta*, aged 7 and 8 years, respectively, and weighing between 5 and 6 kg) renamed M1 and M2. Animal care, housing, and experimental procedures were in compliance with the National Institutes of Health’s Guide for the Care And Use of Laboratory Animals and approved by the Ethical board of Institut de Neurosciences de la Timone (ref. 2016060618508941).

#### Alert monkey fMRI

The monkeys were first scanned for identifying TVAs. All the details about the fMRI procedures are reported in (*16*)—in which the two monkeys were called M2 and M3. Here, we give only a brief description of these details.

Functional scanning was done using an event-related paradigm with clustered-sparse acquisitions on a 3-T MRI scanner (Prisma, Siemens Healthcare), equipped with an 8-channel surface coil (KU, Leuven). Ferrous oxide contrast agent [monocrystalline iron oxide nanoparticle (MION)] was used for all the scanning sessions. Monkeys were trained to stay still in the scanner for a fixed period of 8 s to receive the reward. To avoid interference between sound stimulation and scanner noise, the scanner stopped acquisitions such that three repetitions of 1 of the 96 stimuli (interstimulus interval of 250 ms) were played on a silent background. The 96 stimuli consisted of brief complex sounds from four main categories: human voices, macaque vocalizations, marmoset vocalizations, and nonvocal sounds. Then, MION functional volumes were acquired using echo planar imaging (EPI) sequences (multiband acceleration factor: 2, repetition time, *TR* = 0.955 s). The analysis included 67 MION runs of M1 and 64 MION runs of M2. Voice-selective areas were identified as those regions responding significantly more to conspecific (macaque) vocalizations versus nonvocal sounds (Fig. 1A).

#### fMRI-guided electrophysiology

Monkeys were chronically implanted with high-density microelectrode arrays (CerePort Utah Array, Blackrock Microsystems, Salt Lake City, UT, USA) to record extracellular activity in the fMRI-localized TVAs. Functional maps projected on the individual anatomical surfaces were used to calculate the exact position of the arrays. M1 was implanted with two 32-channel arrays in the anterior TVA (aTVA) of the right rSTG (parcellation from the D99 macaque brain template; Ts2 from the AC map macaque brain template) and one 32-channel array in the right frontal cortex. M2 was implanted with three 32-channel arrays in the left rSTG (Ts2), of which one in the aTVA. In this study, we analyzed only data collected from the arrays implanted in the aTVA (i.e., two arrays for M1 and one array for M2; Fig. 1A).

Electrical signals were amplified and processed using an RZ2 BioAmp Processor (Tucker-Davis Technologies, Alachua, FL, USA) and sampled at 24,414 Hz. Raw data collected during recordings were high-pass filtered (300 to 5000 Hz), and spike sorting was performed offline using the fully automated algorithm MountainSort v5 (*43*) with the SpikeInterface package (*44*). This algorithm detects, for each channel, individual clusters that are then filtered by applying specific thresholds to their quality parameters to identify single neurons (*43*). Clusters with very low FRs (<0.5 Hz) were discarded. Last, the mean waveforms of the remaining clusters were visually inspected to exclude neurons with irregular shapes.

To limit the possible inclusion of the same neuron across sessions, we selected sessions separated in time by at least 3 days [M1: 9 ± 4 (mean ± SD) days’ intervals between sessions on average; M2: 17 ± 8 days’ intervals on average]. Our final dataset was composed of 475 single neurons, with 358 neurons from M1 across 11 recording sessions, and 117 neurons from M2 across 5 recording sessions.

#### Experimental setup and behavioral task

All recordings were performed in an acoustically insulated room. The monkeys sat in a primate chair with the head fixed by a noninvasive modular restriction mask (MRM) developed in our laboratory. Auditory stimuli were presented through an RZ6 Multi-I/O Processor (Tucker-Davis Technologies, Alachua, FL, USA) and transduced by two 8020 Genelec speakers, which were positioned at ear level 72 cm from the head and 60° to the left and right. Stimuli were delivered at a sound pressure level of ~92 dB. Hand detection was achieved using two optical sensors.

Monkeys were trained to perform a pure tone detection task (Fig. 1B). This task was introduced to maintain the attention of the monkeys on the auditory stimulation. They were required to hold a bar with both hands for 1500–2000 ms to trigger the presentation of the sounds. In each trial, from three to seven stimuli (interstimulus interval: 280 to 540 ms) were played after which a 500-ms 1000-Hz pure tone was presented. The pure tone instructed the monkeys to release the bar to receive the juice reward (correct trials). If the monkeys released the bar before the pure tone presentation (false alarm trials) or did not release the bar (miss trials; upper reaction time: 250 ms), no reward was given.

#### Auditory stimuli

During each recording session, we presented multiple sets of stimuli with a fixed order. The stimuli within each set were presented in a randomized order, but all stimuli were presented once before any repetition occurred, ensuring a balanced number of repetitions.

The main set of stimuli (“voice identity”) in this experiment consisted of 25 natural vocalizations of rhesus macaques (kindly provided by M. Hauser). Specifically, we included five “coo calls” from each of the five individuals of macaque (duration time: 300 to 500 ms). The stimuli were collected from subjects that were unfamiliar with the experimental monkeys. Stimuli were resampled at 48,828 Hz and normalized by root mean square amplitude. Last, a 10-ms cosine ramp was applied to the onset and offset of the stimuli.

At the beginning of each recording session, we presented a “voice localizer” stimulus set consisting of 12 macaque vocalizations and 12 nonvocal sounds. Macaque vocalizations (kindly provided by M. Hauser) included different call types (coos, grunts, barks, and screams). Nonvocal sounds included both natural and artificial sounds from previous studies from our group (*45*, *46*) or kindly provided in (*13*) and (*47*). Stimuli were adjusted in duration so that all of them lasted 500 ms.

In each recording session, after the localizer, we also assessed the tonotopic organization of the recorded areas, by means of a “band-passed noise” (BPN) stimulus set consisting of five stimuli, each with a central frequency ranging from 125 to 16,000 Hz, spaced in 1.75 octave steps (band weight: ⅓ octave). The results regarding the tonotopic organization of the aTVA have been previously reported in (*17*), where no clear tonotopic organization was observed in this region.

### Data analysis

#### Spiking data preprocessing and neurons selection

Analyses were conducted using MATLAB (The MathWorks Inc., Natick, MA, USA) and the open-source statistical software R. The dataset for this study comes from recording sessions in which each of the 25 voice identity stimuli was repeated at least 28 times in M1 sessions and 27 times in M2 sessions.

Spike times were saved at a resolution of 1 ms. All the data here were aligned to the onset of sounds. Baseline activity was defined as the average FR during the 100-ms period preceding stimulus onset. To compute *z* scores, the average response to each stimulus was normalized to SD units relative to the baseline. Because the duration of the macaque voice identity stimuli varied between 300 and 500 ms, we analyzed a 300-ms window following stimulus onset, corresponding to the minimum presentation time across all stimuli.

Auditory-responsive neurons were selected by calculating *z*-scored responses to each of the 25 voice identity stimuli in 10-ms bins throughout the stimulus presentation. Neurons were classified as auditory-responsive if their *z*-score exceeded 2 SDs for at least two consecutive bins in response to one or more stimuli [similar to methods from (*14*, *17*)]. Only auditory-responsive neurons (76 from the most anterior array of M1; 97 from the most posterior array of M1; 44 from M2) were included in further analyses.

To compute the mean normalized responses of each neuron to the stimuli in Fig. 1F, we calculated the average response to each stimulus in a 200-ms window after onset, subtracted the baseline, and normalized by the absolute maximum across the stimuli.

#### Voice selectivity index

For each neuron, we calculated a VSI using the localizer stimulus set. The VSI was defined as

$$\text{VSI}=\frac{r_{\text{voice}}-r_{\text{non-vocal}}}{r_{\text{voice}}+r_{\text{non-vocal}}}$$ (1)

where* r*_voice_ is the average neuronal response to macaque vocalizations, subtracted of the baseline, in a 200-ms window following stimulus onset, and *r*_non-vocal_ is the average neuronal response to nonvocal sounds, subtracted of the baseline, in the same time window.

A VSI of 0 indicates equal responses to voices and nonvocal sounds. A VSI of 0.33 indicates a response twice as strong to vocalizations compared to nonvocal sounds, whereas a VSI of −0.33 reflects a response twice as strong to nonvocal sounds as to voices. In cases where *mean*_voice_ > 0 and *mean*_non-vocal_ < 0, the VSI was set to 1; conversely, in cases where *mean*_voice_ < 0 and *mean*_non-vocal_ > 0, the VSI was set to −1 (*5*, *17*).

#### Identity selectivity of neurons

To quantify the identity selectivity of each neuron, we computed a PI that measured how strongly a neuron responded to its preferred identity—defined as the identity eliciting the highest average FR within a 200-ms window following stimulus onset—relative to its responses to all other identities. The PI was calculated as

$$\text{PI}=\frac{n-\left(\frac{\sum r_{i}}{r_{\max}}\right)}{n-1}$$ (2)

where *n* is the number of identities; *r_i_* is the average neuronal response to identity*_i_*, subtracted of the minimum *r_i_*, in a 200-ms window following stimulus onset; and *r*_max_ is the maximum *r_i_*. A value of 0 indicates an identical response to the five identities; a value of 1 indicates modulated discharge for only one identity (*48*).

#### ROC analysis

To test whether each neuron responded selectively and consistently to the vocalizations of a preferred identity, we performed a ROC analysis, following methods from prior studies (*23*, *26*). For each neuron, the true-positive rate (*y* axis) was defined as the proportion of the vocalizations from the preferred identity that evoked a response (mean FR) above a given threshold. The false-positive rate (*x* axis) was defined as the proportion of the remaining vocalizations (from other individuals) that exceeded the same threshold. The ROC curve was obtained by systematically varying the response threshold across its dynamic range. Neurons that responded reliably to all vocalizations from the preferred identity—but not to others—produced a convex ROC curve with an AUC approaching 1, indicating strong identity-invariant coding. In contrast, neurons that responded randomly across all voices yielded ROC curves with AUCs near 0.5, reflecting chance-level performance.

To assess statistical significance, we implemented a nonparametric surrogate test. For each neuron, we generated 99 surrogate ROC curves by randomly selecting five stimuli (i.e., the number of stimuli per identity) from the total of 25 and computing the AUC for each random set. A neuron was considered to exhibit a significant invariant response to its preferred identity if it is true that the AUC exceeded that of all 99 surrogate AUCs (corresponding to a significance level of *P* < 0.01).

To determine how many identities each neuron selectively encoded, we repeated this analysis independently for each of the five individual identities, assessing the presence of significant identity-invariant coding for each.

#### Decoding analysis

We used an MCC classifier—as implemented in the MATLAB neural decoding toolbox (*49*)—to analyze the aTVA neuronal population or different subpopulations of neurons (based on VSI or PI). The classifier was trained to discriminate between the five voice identities.

To train the classifier, trials were labeled on the basis of the identity of the stimulus presented, and FRs from trials and neurons were binned in 20-ms sliding windows, every 10-ms bins. We tested 44 consecutive bins, from −100 ms (i.e., a time window from −100 to −80 ms) to +330 ms from the stimulus onset. Note that these 20-ms bins are plotted such that the decoding accuracy is aligned to the center of each bin. For each bin, a different classifier was trained/tested.

*z* score normalization was applied to each neuron, to give equal weight to all the units regardless of FR. Then, the classifier was trained using a *k* cross-validation splits procedure, where *k* represents the maximum number of available trials for each condition for each neuron. In particular, the classifier was trained using *k* − 1 splits and then tested on the remaining split. All possible train/test splits were tested and this process was repeated 50 times (i.e., 50 runs) with different subsets of trials. The classification accuracy from these runs was then averaged.

To account for differences in population size when comparing classification accuracy between neuronal subpopulations of unequal size (based on VSI or PI), we computed the average (±SE) classification accuracy for the larger subpopulations by generating 100 random subsamples, each containing *n* neurons, where *n* matched the size of the smaller subpopulation.

To assess whether the obtained decoding accuracies were above chance, we ran a permutation test that consisted of repeating the full decoding procedure 50 times with the labels of identities randomly shuffled. We obtained a null distribution of shuffled data, and the decoding results were considered significantly above chance if they were greater than all the shuffled data in the null distribution [*P* value threshold of *P* = 1/(50 * 44) = 0.0004]. The latency of when the *P* values are first above chance corresponds to the first time bin of three consecutive bins with *P* values below the *P* value threshold.

To determine the similarity between two classification accuracy distributions, we computed for each time bin a distribution-free overlapping index (η) using the overlapping package for R (*50*). The overlapping index η represents the proportion of the overlapping area between the probability density functions of two distributions. In this sense, an overlapping index of η(A,B) = 0 indicates that f_A (X) and f_B (X) are distinct. Two distributions were considered as significantly different for η < 0.05 for at least three consecutive time bins.

For the high-identity-selective and low-identity-selective subpopulations, we also performed a cross-temporal decoding analysis to establish the temporal evolution of information coding as previously done (*51*–*53*). This analysis results in a classification accuracy matrix where the values along the diagonal are calculated by performing training and testing on equivalent time bins. In contrast, different time bins for training and testing are used to calculate the off-diagonal values. We classified the stability of off-diagonal time points by implementing the method used in (*53*).

#### Dimensionality reduction approach

To investigate how neural population responses evolve over time and reflect voice identity, we applied PCA to reduce the dimensionality of the neuronal response space and visualize stimulus-evoked activity as trajectories in a low-dimensional state space.

For each neuron and stimulus, we computed spike density functions (SDFs) in 1-ms bins, convolved with a Gaussian kernel (σ = 10 ms), over a window spanning −100 to +300 ms relative to stimulus onset. SDFs were further smoothed using a 100-ms sliding window and then organized into matrices per stimulus, where each row corresponded to a neuron. These matrices were concatenated across stimuli along the time dimension, preserving neuron identity [i.e., final matrix: N_neurons × (stimuli × time bins)]. Neuronal activity was *z*-scored (mean subtracted and divided by SD) across the concatenated matrix to normalize across units. PCA was then applied to this matrix, and the first three principal components were used to define a reduced-dimensional neural state space.

To assess whether trajectories in this space clustered according to voice identity, we computed Euclidean distances between trajectories. Specifically, we averaged the pairwise distances (*n* = 50) between trajectories corresponding to the same identity (Within IDs distance). Similarly, we averaged the pairwise distances (*n* = 250) between trajectories from different identities (Between IDs distance). Statistical comparisons between the “Within IDs distance” and “Between IDs distance” were performed using bootstrapped two-sample *t* tests (100,000 iterations, one-tailed), with Bonferroni correction applied for multiple comparisons, resulting in a corrected significance threshold of *P* = 0.0001 (corresponding to *P* = 0.05/401 time bins).

#### Representational similarity analysis

We performed RSA to compare neuronal data with acoustic data and evaluate theoretical models of voice identity representation. In the first analysis, we computed a single 25 × 25 Neuronal RDM for each of the 10 subpopulations of neurons—five groups of high-identity-selective neurons and five groups of low-identity-selective neurons—defined by their preferred identity. For each group, population responses were extracted as vectors of *z*-scored average FRs for each of the 25 voice identity stimuli in the 0- to 200-ms poststimulus window. Pairwise dissimilarities between stimuli were calculated using Euclidean distances, resulting in ten Neuronal RDMs. Each was compared to one of the five Model RDMs, each representing the idealized structure in which one identity is maximally dissimilar from all others. Planned comparisons involved analyzing between-identities versus within-identity portions of Neuronal RDMs, predicted by the models, using bootstrapped two-sample *t* tests (100,000 iterations, one-tailed; Bonferroni-corrected *P* value threshold: *P* = 0.05/50 = 0.001).

In a second analysis, we generated time-resolved Neuronal RDMs to examine the temporal dynamics of voice identity encoding. For each of the 25 stimuli, we extracted vectors of *z*-scored average FRs in 10-ms bins using a ±25-ms sliding window from −100 to +300 ms relative to stimulus onset. At each time bin, we computed a 25 × 25 RDM using Euclidean distances between stimulus-evoked population vectors, resulting in a sequence of 41 Neuronal RDMs.

We compared each time-resolved Neuronal RDM with two reference matrices: (i) a Model RDM capturing the ideal pattern of Discrimination (maximal dissimilarity between identities) and Recognition (minimal dissimilarity within identities) and (ii) an LTAS RDM representing pairwise dissimilarities in LTAS, to account for low-level spectral differences among stimuli. We applied two RSA paradigms:

1) In Paradigm 1, we computed Spearman’s rank correlations between the upper triangles (excluding diagonals) of the 41 Neuronal RDMs and the Model RDM (Bonferroni corrected *P* value threshold of *P* = 0.05/41 = 0.0012).

2) In Paradigm 2, we repeated this analysis while partialing out the LTAS RDM—using the partialcorr Matlab function—to isolate the contribution of higher-level identity representations.

Last, we tested the model-predicted Discrimination and Recognition components of the Neuronal RDMs by extracting the corresponding matrix portions and comparing them via bootstrapped two-sample *t* tests (*P* < 0.0012). Each curve was also compared against baseline activity (−100 to 0 ms) using paired-sample *t* tests with the same correction.

#### Reverse correlation analysis of neuronal contributions to model alignment

To assess each neuron’s contribution to the Discrimination and Recognition processes, we adapted a reverse correlation approach inspired by the “bubbles” method (*54*, *55*). For this analysis, we used the following two Model RDMs: (i) Discrimination Model as a categorical matrix where stimuli from different identities are maximally dissimilar and those from the same identity are similar and (ii) Recognition Model as a matrix emphasizing one identity, with minimal within-identity dissimilarity and maximal dissimilarity to other identities.

We first randomly selected 30 neurons from the total population of 217 and computed a Neuronal RDM based on their activity in the 0- to 200-ms poststimulus window. This procedure was repeated 20,000 times. The alignment between each Neuronal RDM and each Model RDM was quantified using a similarity index

$$\text{Similarity score}=1-d\left({\mathrm{RDM}}_{\text{neuronal}}-{\mathrm{RDM}}_{\text{model}}\right)$$ (3)

where *d* is the mean difference between RDMs. This index approaches 1 when the Neuronal RDM closely matches the Model RDM. This yielded an inclusion matrix (20,000 × 217) indicating whether each neuron was included in each sample and a vector of 20,000 similarity scores.

For each neuron, we then computed a Pearson correlation between its binary inclusion vector and the similarity scores. This yielded an individual contribution index, with values near 1 indicating strong positive influence on alignment with the model, values near 0 indicating no contribution, and negative values indicating inverse alignment. This procedure generated two indices per neuron: a Discrimination Index, based on alignment with the Discrimination Model, and a Recognition Index, computed across the five identities by correlating neuron inclusion with identity-specific recognition scores. In this case, the maximum correlation across identities was retained to capture each neuron’s strongest recognition-related contribution, regardless of identity.

## Supplementary Materials

### The PDF file includes:

Figs. S1 to S5 Legends for audio S1 to S4

### Other Supplementary Material for this manuscript includes the following:

Audio S1 to S4
