# Supplementary material for: Voice identity invariance by anterior temporal lobe neurons
Source: Sci Adv. 2025 Aug 29;11(35):eadv7033. doi: 10.1126/sciadv.adv7033 (PMC12396339; doi:10.1126/sciadv.adv7033)
Supplement: Supplementary file 1 — Figs. S1 to S5 Legends for audio S1 to S4 [file sciadv.adv7033_sm.pdf]

Science Advances
AAAS

Supplementary Materials for

Voice identity invariance by anterior temporal lobe neurons

Margherita Giamundo *et al.*

Corresponding author: Margherita Giamundo, margherita.giamundo@univ-amu.fr;
Pascal Belin, pascal.belin@univ-amu.fr

Sci. Adv. **11**, eadv7033 (2025)
DOI: 10.1126/sciadv.adv7033

The PDF file includes:

Figs. S1 to S5
Legends for audio S1 to S4

Other Supplementary Material for this manuscript includes the following:

Audio S1 to S4

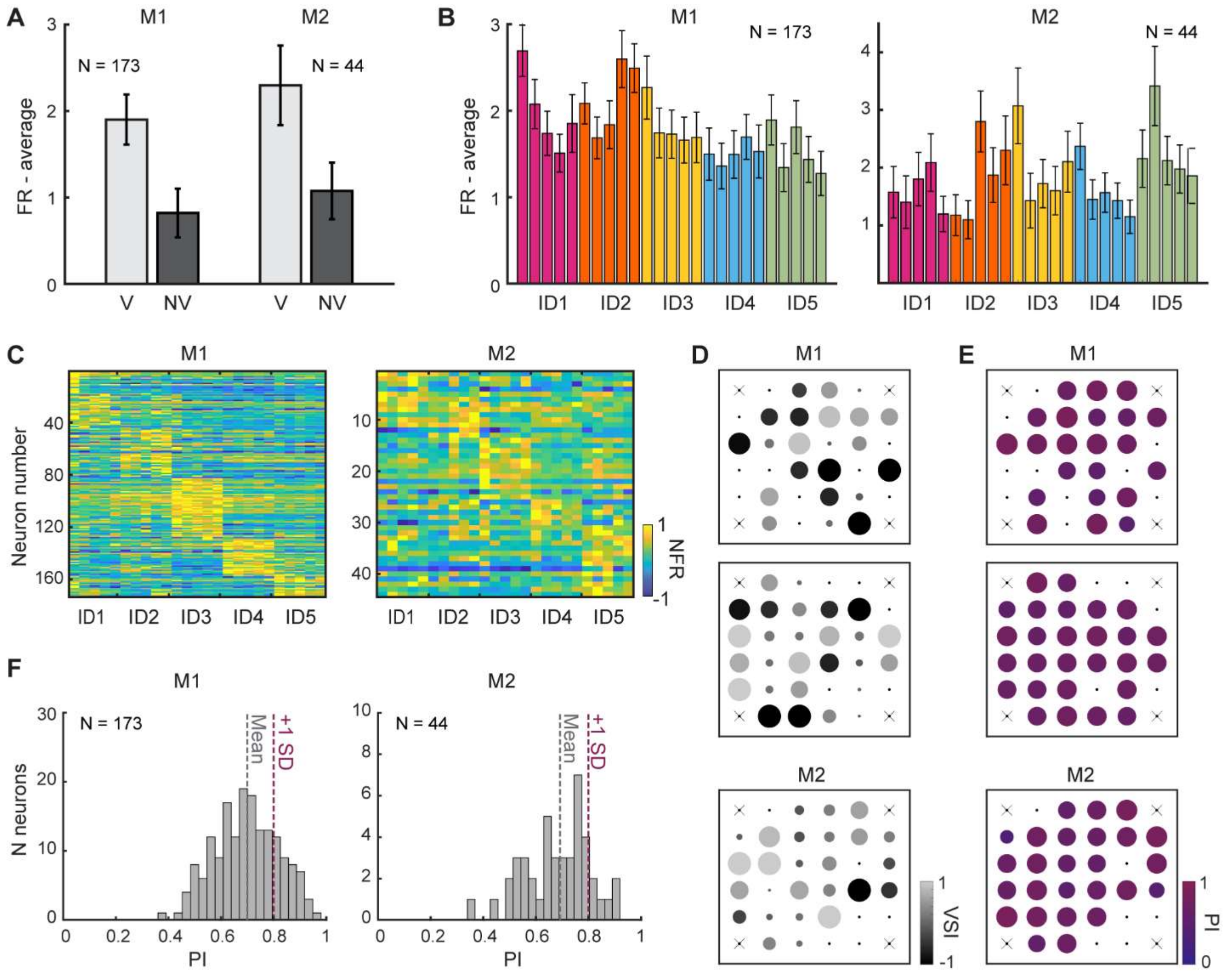

Fig. S1. Responsiveness of aTVA neurons to voice identity in Monkey 1 (M1) and Monkey 2 (M2).

(**A**) Average population response (mean ± SE) to macaque vocalizations (V) and non-vocal sounds (NV) of the localizer stimulus set. (**B**) Average population response to the 25 voice identity stimuli, with the color corresponding to each identity. (**C**) Neurons responsiveness to the 25 voice identity stimuli. Each row represents the average response of a single neuron across the 200 ms post-stimulus onset. Neurons are sorted based on the identity eliciting their strongest response. (**D-E**) Representation of Voice Selectivity Indices (VSIs) contrasting macaque vocalizations vs. non-vocal sounds (**D**) and of Preference Indices (PIs) (**E**) in electrophysiological recording sites. Index values were averaged across neurons recorded at each electrode of the arrays. Dot size reflects the absolute value of the corresponding index. (**F**) Histograms of PIs, reflecting each neuron's selectivity for a single identity. A PI of 0 indicates equal responsiveness across all five identities, while a PI of 1 reflects exclusive responsiveness to a single identity. Vertical dashed lines represent the mean PI (Mean) and the mean plus one standard deviation (+1 SD).

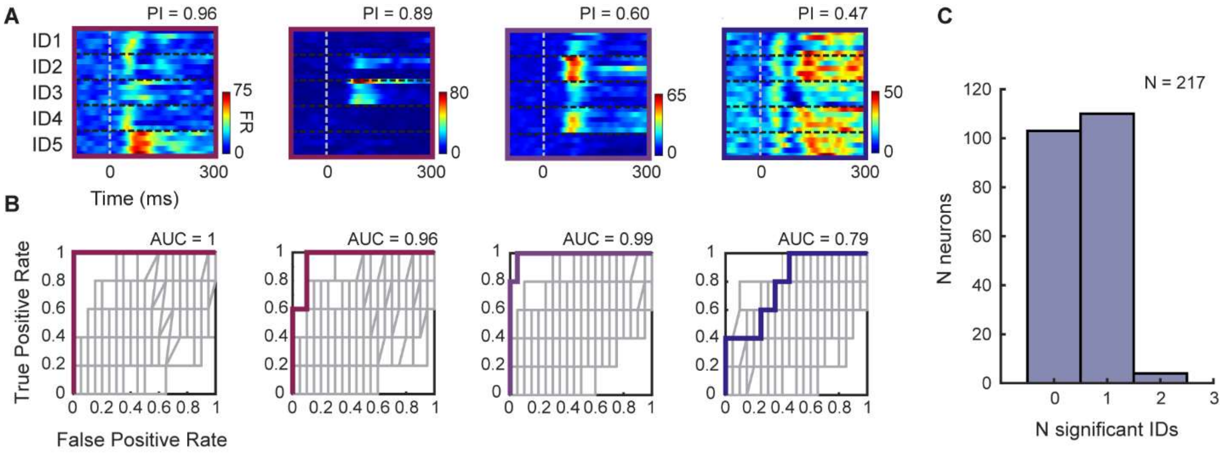

Fig. S2. Examples of aTVA neurons modulated by voice identity.

(**A**) Mean response time courses of four representative neurons to the 25 voice identity stimuli. Each group of five rows corresponds to the five coo calls from a single individual (e.g., the first five rows represent ID1). Responses are aligned to stimulus onset. (**B**) Corresponding ROC curves (color traces) testing whether each neuron responded in an invariant manner to the five voices of its preferred identity (true positive rate) but not to other voices (false positive rate). Gray traces show 99 surrogate ROC curves computed from randomly selected voice sets ($p < 0.01$). (**C**) Histogram showing the number of identities to which each neuron was selectively responsive, based on the ROC analysis. Most neurons showed selective, invariant responses to a single identity, with a small proportion responsive to two.

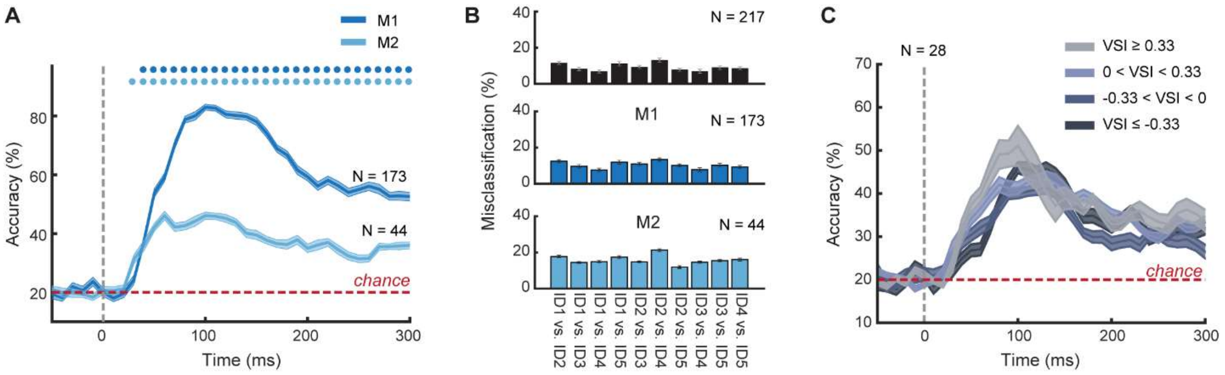

Fig. S3. Encoding of voice identity information by neuronal populations.

(**A**) Time-resolved classification accuracy (mean ± SE) of linear classifiers trained to discriminate between five individual identities (chance level = 20%), based on the spiking activity from neurons recorded in monkeys M1 and M2. Colored dots indicate time bins with significantly above-chance classification accuracy (permutation tests, $p < 0.0004$). (**B**) Misclassification rates for all identity pairs, computed using neurons from both monkeys (top), M1 only (middle) and M2 only (bottom). (**C**) Identity classification accuracy for four subgroups of neurons defined by their VSI. To refine this analysis, neurons with VSI = 1 or VSI = -1 (values assigned by convention when comparing normalized firing rates with opposite signs; see Methods) were excluded. To control for group size, the decoding curves for the voice-selective group (VSI ≥ 0.33; N = 38) and for neurons with weak positive voice selectivity (0 < VSI < 0.33; N = 36) were averaged from 50 random subsamples of 28 neurons, matching the sample size of the voice-inhibited group (VSI ≤ -0.33; N = 28) and the group with weak negative selectivity (-0.33 < VSI < 0; N = 28).

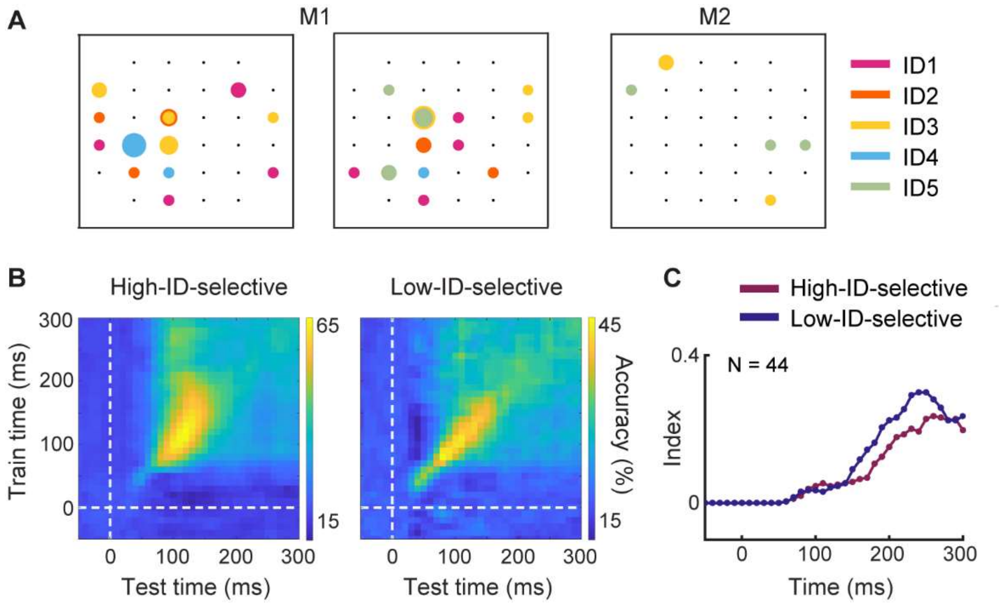

Fig. S4. Encoding of voice identity in high- versus low-identity-selective neurons.

(**A**) Spatial distribution of high-identity-selective neurons (N = 44) across electrophysiological recording sites. Dot size reflects the number of high-identity-selective neurons recorded at each site; colors indicate preferred identity based on tuning profiles. (**B**) Cross-temporal decoding accuracy for high- and low-identity-selective subpopulations. The y-axis indicates the time bins used to train the classifier, and the x-axis shows time bins used for testing. A diagonal band of high accuracy reflects dynamic coding, in which population responses rapidly change over time. Broader square-like regions indicate static coding, with a stable representation across time. (**C**) Stability indices for each subpopulation, quantifying the stability of the coding over time. High stability index values indicate stronger temporal stability in neural coding.

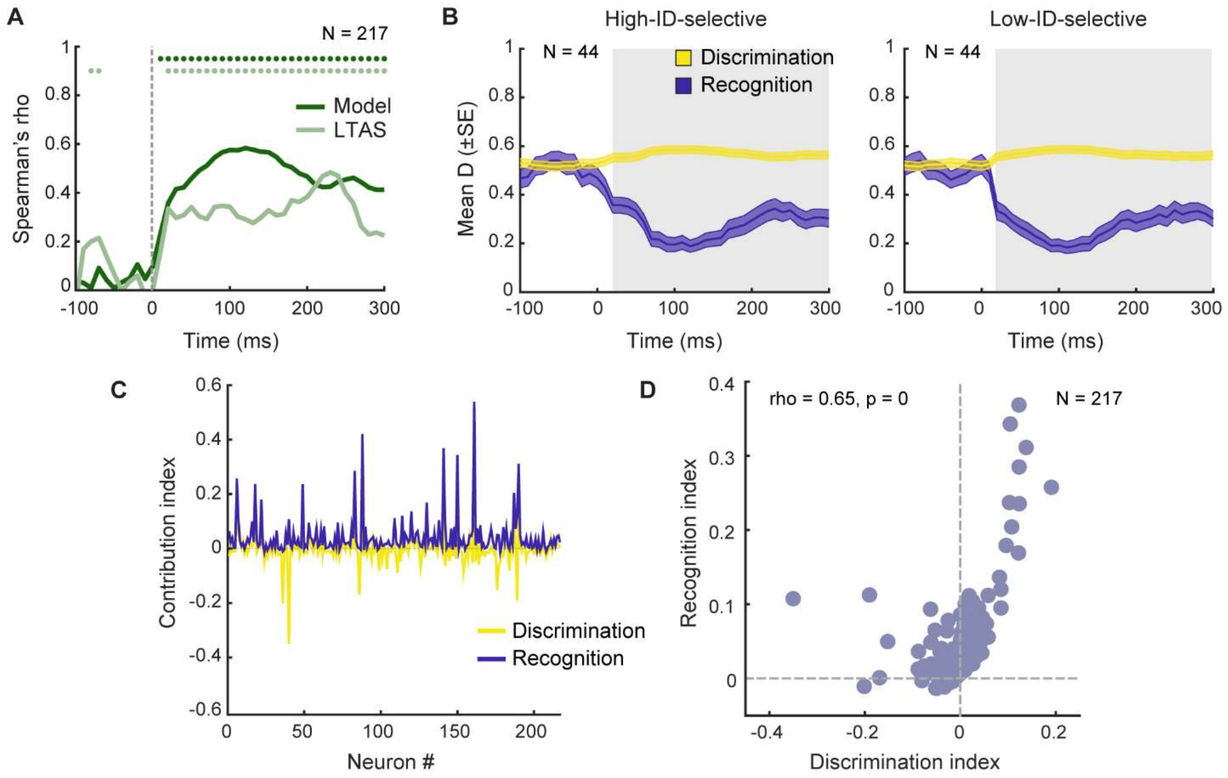

Fig. S5. Model-based analysis of voice identity coding in aTVA neurons.

(**A**) Time course of correlations (Spearman) between time-resolved Neuronal RDMs with the Model RDM or long-term average spectrum (LTAS) RDM. Colored dots indicate time bins with significant correlations (Bonferroni-corrected $p < 0.0012$). **(B)** For high- and low-identity-selective subpopulations, dissimilarity values (mean ± SE) of the portions of Neuronal RDMs corresponding to Discrimination (yellow curve) and Recognition (blue curve) processes, as predicted by the theoretical model. Gray shaded area indicates time bins of statistically significant association with the theoretical model (bootstrapped two-sample t-tests, Bonferroni-corrected $p = 0.0012$). **(C)** Each neuron's contribution to Discrimination and Recognition processes. Contribution indices were computed for each neuron, quantifying its influence on the match between Neuronal RDMs and either the Discrimination or Recognition model RDMs (see Methods). Values near 1 indicate strong positive alignment, near 0 indicate no contribution, and negative values indicate an inverse relationship. **(D)** Correlation between the Discrimination and Recognition contribution indices across the neuronal population. Most neurons contributed to both processes, although, on average, the Recognition contribution index was higher than the Discrimination index.

Audio S1-S4.

Representative examples of four coo calls, with two calls produced by each of two different macaques.
